# A Smart Drug Delivery System Based on Biodegradable Chitosan/Poly(allylamine hydrochloride) Blend Films

**DOI:** 10.3390/pharmaceutics12020131

**Published:** 2020-02-04

**Authors:** Muhammad Sohail Sarwar, Qingrong Huang, Abdul Ghaffar, Muhmmad Amin Abid, Muhammad Sohail Zafar, Zohaib Khurshid, Muhammad Latif

**Affiliations:** 1 Department of Chemistry, University of Engineering and Technology, Lahore 54890, Pakistan; sohailchemist77@gmail.com; 2 Department of Food Science, Rutgers, The State University of New Jersey, New Brunswick, NJ 08901, USA; qhuang@sebs.rutgers.edu; 3 Department of Chemistry, Forman Christian College (A Chartered University), Lahore 54600, Pakistan; 4 Department of Chemistry, University of Sahiwal, Sahiwal 57050, Pakistan; mabiduet@gmail.com; 5 Department of Restorative Dentistry, College of Dentistry, Taibah University, Al Madinah, Al Munawwarah 41311, Saudi Arabia; MZAFAR@taibahu.edu.sa; 6 Department of Prosthodontics and Dental Implantology, College of Dentistry, King Faisal University, Al-Ahsa 31982, Saudia Arabia; drzohaibkhurshid@gmail.com; 7 Centre for Genetics and Inherited Diseases (CGID), Taibah University, Madinah Al Munawwarah 42318, Saudi Arabia

**Keywords:** drug delivery platform, chitosan, natural and synthetic materials in drug delivery, biodegradation, polymers, Ciprofloxacin, blend, film, anti-infective agents

## Abstract

The amalgamation of natural polysaccharides with synthetic polymers often produces fruitful results in the area of drug delivery due to their biodegradable and biocompatible nature. In this study, a series of blend films composed of chitosan (CS)/poly(allylamine hydrochloride) (PAH) in different compositions were prepared as smart drug delivery matrices. The properties of these polymeric films were then explored. Attenuated total reflectance-Fourier transform infrared (ATR-FTIR) analysis confirmed an intermolecular hydrogen bonding between CS and PAH. Atomic force microscopy (AFM) revealed improvements in surface morphology as the percentage of PAH in the blend films increased up to 60% (*w*/*w*). Water contact angle (WCA) ranged between 97° to 115°, exhibiting the hydrophobic nature of the films. Two films were selected, CTH-1 (90% CS and 10% PAH) and CTH-2 (80% CS and 20% PAH), to test for in vitro cumulative drug release (%) at 37 ± 0.5 °C as a function of time. It was revealed that for simulated gastric fluid (SGF) with pH 1.2, the cumulative drug release (CDR) for CTH-1 and CTH-2 was around 88% and 85% in 50 min, respectively. Both films converted into gel-like material after 30 min. On the other hand, in pH 7.4 phosphate buffer saline (PBS) solution, the maximum CDR for CTH-1 and CTH-2 was 93% in 90 min and 98% in 120 min, respectively. After 120 min, these films became fragments. Sustained drug release was observed in PBS, as compared to SGF, because of the poor stability of the films in the latter. These results demonstrate the excellent potential of blend films in sustained-release drug delivery systems for hydrophilic or unstable drugs.

## 1. Introduction

Natural biopolymers, especially polysaccharides, have attracted considerable interest in the field of drug delivery due to their biodegradable, biocompatible, hydrophilic, and protective properties [1,2,3]. In pharmaceutical research and applications, polymeric nano-particles and natural biological macromolecules-based delivery systems are commonly used these days. These potential carriers are capable of delivering bioactive compounds to specific cells and tissues with minimal immune response at the nanoscale. Natural polymers, due to their diverse physico-chemical properties, can be used to fabricate polymeric nano-carrier systems (NCS) that can optimize the bioavailability of drugs by increasing retention time, minimizing side effects, increasing drug solubility, and reducing dosage frequency [4].

Chitosan, a natural carbohydrate, is inexpensive, easily available, a cationic copolymer, and can be obtained by deacetylation of chitin. It consists of *N*-acetyl-d-glucosamine and d-glucosamine units. Considering the non-toxic, biocompatible, and biodegradable nature, chitosan is a prospective candidate in the field of pharmaceutical industry [4,5]. Several researchers have investigated chitosan in gene delivery, drug release, and biomedical areas [6,7,8,9]. It can increase the extent of drug delivery across mucosal or nasal layers without causing any damage to the tissues [10]. On the contrary, various processing conditions (such as a change in temperature, etc.) and environmental factors may trigger degradation and deteriorate its polymeric structure [11].

It has been observed recently that two or more polymers are usually blended to obtain a wide range of physico-chemical properties. Numerous properties of chitosan can be enhanced by blending with natural polymers as well with synthetic ones such as zein, sodium alginate, curdlan, konjac glucomann, poly(lactic acid), polycaprolactone, poly(ethylene oxide), poly(vinyl pyrollidine), and graphene oxide [12,13,14,15]. Depending on the nature of biomedical applications, such as tissue engineering and drug delivery, chitosan has been blended with polyethylene glycol fumarate, poly(ethylene glycol) methyl ether, bone, polyethylene glycol, cartilage, skin, gelatin, dialdehyde starch, and nerves [14,16,17,18,19,20].

Poly(allylamine hydrochloride) is a water-soluble weak-base, a cationic and biodegradable polymer. It has a large number of amino groups that are present as a free amine or cationic ammonium salt like other amine group-containing polymers [21,22,23]. Chitosan alone cannot satisfy biomedical applications due to mechanical defects; therefore, blends of chitosan are employed to overcome insufficient mechanical features [24,25,26,27,28]. Multitudinous systems comprising of polymeric materials have been prepared to serve as drug carriers to investigate the controlled release behavior of payloads and offer smart drug delivery. Polymeric biocompatible materials such as polyelectrolytes nanoparticles coated with a bilayer of polyelectrolytes, namely PAH and poly (sodium 4-styerenesulfonate) (PSS) were investigated for hydrophobic drug release [29]. It has been reported that PAH and polyurethane multilayer films as a drug delivery system are potential contestants for pharmaceutical and biomedical applications [22]. The efficiency of nanoparticulate drug delivery systems can be increased by modifying poly(d,l-lactide-*co*-glycolide) via layer-by-layer adsorption, with two polyelectrolyte pairs such as PAH/PSS and poly(l-lysine hydrobromide)/dextran sulfate [30].

Several efforts to develop functional materials (films, beads, and hydrogels) from chitosan have been reported for potential use in wound healing [31] and drug delivery systems [32,33,34,35,36,37]. Blending of natural polymers with synthetic polymeric material is an alternative approach to incorporate a desired set of physico-mechanical characteristics in new materials. The film matrix generated by the blending of two or more different polymeric systems generally results in modification of physico-mechanical properties, compared to a film obtained by an individual polymer. For instance, chitosan/starch blend films show higher flexibility and improved elongation [38], chitosan and quaternized poly(4-vinyl-*N*-butyl) pyridine demonstrate robust tensile strength [39], blends of chitosan/poly(vinylalcohol) [40], chitosan/*N*-methylol nylon 6 [41], and chitosan/polycaprolactone [12] exhibit improved properties compared to pure chitosan films. Several publications related to the blending of chitosan with natural (particularly cellulose and its derivative) [42,43,44] and synthetic polymers [11,41,45,46,47] have been reported.

In continuation of our efforts to develop materials for controlled drug delivery [48,49], the purpose of the current study was to develop a smart drug delivery system for ciprofloxacin hydrochloride monohydrate (CPX) using biodegradable CS/PAH blend films. CS and PAH were selected to prepare a series of blend films with different compositions as smart drug delivery matrices. The prepared polymeric films were characterized to explore their properties. To study the drug release behaviour of CS/PAH blend films, CPX was taken as a model drug. CPX is a fluoroquinolone drug that has a broad antibiotic spectrum. It is widely used for microbial infections such as pulmonary, urinary, and dermal infections [50] and anterior ocular infections [51]. It can be administered intravenously or orally [52]. It is very effective against gram-positive and gram-negative bacteria that cause gastrointestinal, urinary tract, abdominal, and respiratory infections. Prophylaxis and osteomyelitis, which is caused by P. aeruginosa, has been treated with CPX [53]. Due to its low solubility, CPX is usually formulated as a colloidal drug carrier, such as micelles, nano-suspensions, and polymeric nanoparticles [54]. CPX offers bioavailability (60–80%) and serum half-life (~3–4 h); however, in clinical settings, repeated administration (b.i.d, for 5 days) concerns have made CPX a suitable candidate for controlled-release drug delivery system that can reduce gastric irritation and dose dumping concerns [55,56]. A number of studies have shown the incorporation of CPX in polymeric matrices and have demonstrated control release to function as wound-dressing materials [57,58,59], and antibacterial efficacy of CPX-loaded imprinted hydrogels [60]. To further address the limitations of instantaneous release of CPX, the aim of this study was to design and develop blend films using a biocompatible and biodegradable polymeric blend of CS/PAH in varying compositions, to be used as a drug delivery matrix for sustained release.

We previously reported drug releasing films prepared by blending CS with methoxy polyethylene glycol (mPEG) for controlled drug release applications [20]. In the present study, based on the well-known fact that cationic polymers have the potential to adhere to negatively charged surfaces of bacteria, we incorporated PAH primarily to impart flexibility in blend films and speculated on the retention of the overall cationic nature of blend films. To the best of our literature survey and knowledge, there are no publications reporting controlled release behaviour of CPX from CS/PAH blended films as potential biomaterials for drug delivery applications.

## 2. Materials and Methods 

### 2.1. Materials

Chitosan (Cat No. 448869, viscosity: 20–300 cP) with deacetylation degree (DDA) value ~75 to 85% molecular weight: 50 to 190 kDa was purchased from Sigma Aldrich, Saint Louis, MO, USA. Poly(allylamine hydrochloride (PAH) (formula weight: 120,000~200,000 g·mol^−1^ was obtained from Beantown Chemical, Hudson, NH, USA. Acetic acid (>99.9%) was also purchased from Sigma Aldrich, Saint Louis, MO, USA. Ciprofloxacin hydrochloride monohydrate was collected from Tokyo Chemical Industry Co. Ltd. (TCI, Portland, OR, USA). All of the other chemicals were used as received and without any further purification.

### 2.2. Preparation of Films

Polymeric blend films of chitosan (CS) with Poly(allylamine hydrochloride) (PAH) were prepared by solution casting technique as reported earlier [61]. The solutions in different CS/PAH ratios (100/0, 90/10, 80/20, 70/30, 60/40, 40/60) were prepared and each film was coded as CTH-0, CTH-1, CTH-2, CTH-3, CTH-4, CTH-5, respectively. In the first step, chitosan was dissolved in acetic acid solution, 2% (*v/v*), with constant stirring for 90 min at room temperature. Secondly, an aqueous solution of PAH, prepared in deionized water, was added in a dropwise manner into the chitosan solutions with continuous stirring at room temperature. These blended solutions were stirred at room temperature for 90 min to obtain homogenous polymeric solutions. For film casting, the polymeric blended solutions were poured into polystyrene petri dishes and allowed to dry under ambient conditions. The blend films were peeled off and immersed in a solution of NaOH (1%), followed by thorough washing with deionized distilled water. Finally, the prepared blended films were placed in an oven at 48 ± 2 °C for 2 days, enclosed in airtight plastic bags, and stored at room temperature until further analysis.

### 2.3. Attenuated Total Reflectance-Fourier Transform Infrared (ATR-FTIR) Spectroscopy

For intermolecular determinations, ATR-FTIR spectra of the prepared films as well as PAH were recorded using a spectrometer (Thermo Electron Corp., Madison, WI, USA). The spectra were taken in transmittance mode ranging from 4000 to 400 cm^−1^ at a resolution of 4 cm^−1^ and 256 scans per sample.

### 2.4. Atomic Force Microscopy (AFM) Studies 

Surface characterization of prepared films was subjected to AFM using Nanoscope IIIa Multimode microscope (Veeco Instruments, Inc., Santa Barbara, CA, USA), equipped with silicon RTESP7 cantilever (Veeco Nanoprobe, Camarillo, CA, USA) in tapping mode. For silicon tip, nominal spring constant of 40 N/m was applied. AFM images were recorded as 10 μm × 10 μm using scan rate of 0.5 Hz in air keeping ambient conditions. For AFM, blended solutions of polymers were spin-coated on newly cleaved mica surface using a spin coater (Laurell Technologies Corporation, North Wales, PA, USA). The spinning rate was kept at 1500 RPM for 2 min. All the prepared samples were placed in the oven at 48 ± 2 °C for two days to obtain complete dried films [13].

### 2.5. Water Contact Angle (WCA) Measurements

To calculate WCA in degree, the dried films were attached to the microscope glass slides using double-sided tape. A water droplet (8 μL) was placed on each film by a micro-injector syringe (Hamilton Company, Reno, NV, USA). Different images were recorded until WCA was constant using video contact angle instrument VCA optima (AST Products, Inc., Billerica, MA, USA). An automatic WCA measurement, from both the right and left-hand side of the drop, was done using VCA-optima software. On average, twenty measurements were performed per film, which was cut from four different sites.

### 2.6. Swelling Behaviour of Blend Films

The swelling experiments of pristine CS film and their blend films with PAH were conducted in deionized water and ionic solutions, such as 0.1 M, 0.3 M, 0.5 M, 0.7 M, 0.9 M, 1.0 M NaCl, and CaCl_2_ by following previously reported procedure [62]. All vacuum dried films were cut into smaller pieces, weighed (~20 mg), and dipped into glass vials containing 40 mL of solvent/solution. Every 20 min, excess solvent was discarded, each vial was dried using tissue paper, and the weight of swollen film was measured along with the vial. This procedure was repeated until equilibrium was achieved. The experiment was repeated thrice for each film to calculate the standard deviation (SD). Degree of swelling was calculated using Equation (1):

(1) $$\mathrm{Swelling}\;\left(g/g\right)=\frac{W_{s}-W_{d}}{W_{d}}$$

*W*_d_ = weight of the dried films, *W*_s_ = weight of the swollen films.

### 2.7. Cumulative Drug Release (CDR) Study

#### 2.7.1. Preparation of Simulated Gastric Fluid (SGF) and Phosphate Buffer Saline (PBS) Solution

SGF (pH 1.2) was prepared by mixing NaCl (1 g) with 4 mL of HCl and diluted to 500 mL using deionized water [62]. PBS (pH adjusted to 7.4 using 0.1 M NaOH and 0.1 M HCl) solution was prepared by dissolving NaCl (8 g), KCl (0.2 g), Na_2_HPO_4_ (1.44 g), and KH_2_PO_4_ (0.24 g) in 800 mL deionized water and diluted to 1000 mL [63]. A pH meter (Fischer Scientific accumet, Singapore 139949, Singapore) was used to monitor the pH of solutions.

#### 2.7.2. Drug Loading Method

To study controlled drug release, 20 mg of CPX was dissolved in deionized water. The drug solution was added dropwise in a polymeric blended solution of CS/PAH (90/10). The solution was stirred for 90 min at room temperature prior to pouring into a polystyrene petri dish for film formation under ambient conditions. CPX was also loaded in a film with CS/PAH (80/20) by repeating the same procedure as mentioned above. Finally, the prepared blended films were peeled off, enclosed in airtight plastic bags, and stored at room temperature in the dark until further analysis.

#### 2.7.3. In Vitro Drug Release

The CDR mechanism was studied using a smart dissolution tester (Sortax AG, CH-4123, Allschwil 1, CE 7smart, Basel, Switzerland) [64]. The average thickness of drug-loaded films was 40–70 µm, determined using a micrometre (Mitutoyo Corporation, model PK-0505CPX, Kanagawa, Japan) by performing three measurements for each specimen. Films were cut from six different regions for triplicate measurements in SGF and PBS solutions. The average weight of the drug-loaded CTH-1 and CTH-2 films was 0.0791 g and 0.0588 g, respectively. These drug-loaded films were put into a 500-mL dissolution medium, such as SGF and PBS solution, at temperature 37 ± 0.5 °C and stirred at 50 rpm. Every 10 min, 5 mL solution was collected separately from each SGF and PBS solution using a pipette. The obtained solutions were filtered through a membrane (pore size; 0.45 μm). Fresh 5 mL of the SGF and PBS solutions were added back every time to keep the volume at 500 mL. The drug release analysis was investigated for 2–3 h in the SGF and PBS solutions. UV scans of the collected solutions were recorded at *λ*_max_ 277 nm for SGF and 270 nm for PBS using a UV–Visible spectrometer (Agilent Technologies, Cary 60, UV–Vis, Santa Clara, CA, USA). The standard drug solutions of CPX (100 ppm) in SGF and PBS were used as a reference [62].

### 2.8. Statistical Analysis

Data were handled using the SPSS (v.20, IBM, USA) and expressed in the form of means ± standard deviation (SD). The data were analyzed statistically by the two-way analysis of variance (ANOVA) and *p* < 0.05 was considered as statistically significant. Independent sample t-test was applied to compare drug release in different two media, SGF and PBS.

## 3. Results and Discussion

### 3.1. Preliminary Characterization

**Figure 1:**
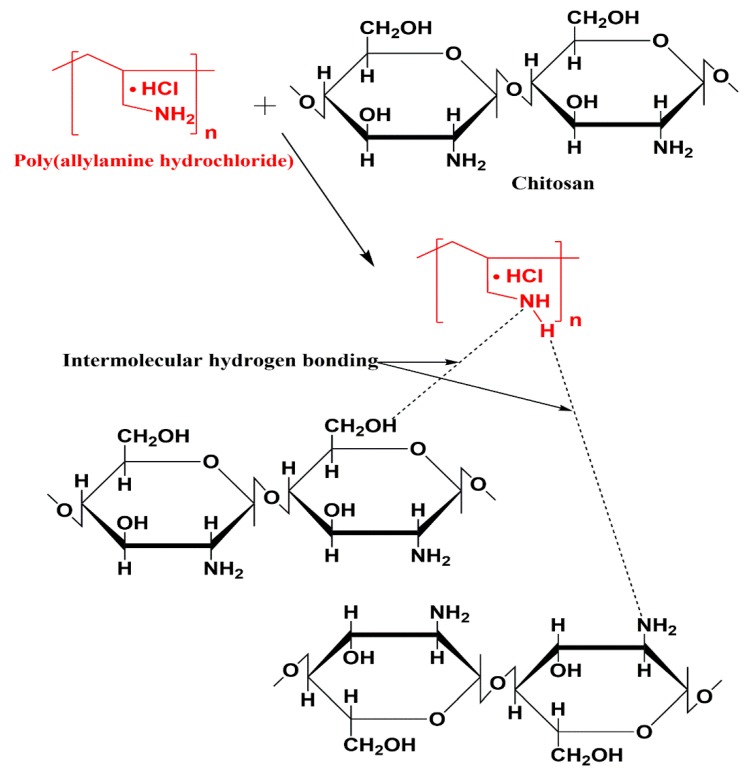
The proposed interactions between the CS and PAH in the prepared blend films.

We prepared polymeric blend films of CS/PAH using the solution casting technique. A solution of chitosan in acetic acid (2% *v/v*) and an aqueous solution of PAH were prepared separately. The aqueous solution was then added drop by drop to the chitosan solution. A preliminary examination showed that the prepared films were translucent and slightly yellowish in color. Films thickness (~40–70 µm) was determined using a micrometre (Mitutoyo Corporation, model PK-0505CPX, Kanagawa, Japan) by performing three measurements per specimen (Table 1). We could not find any statistically significant difference in film thickness among the six samples (CTH0-CTH5) using ANOVA, F(5,18) = 2.35, *p*-value = 0.083. The projected interactions between CS and PAH are shown in Figure 1.

### 3.2. ATR-FTIR Spectroscopy

**Figure 2:**
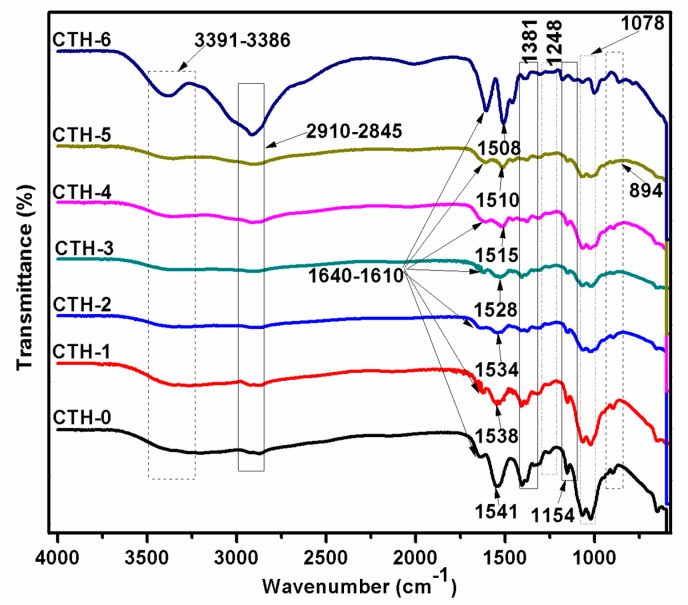
ATR-FTIR spectra of chitosan (CTH-0), PAH (CTH-6), and their blend films showing different chitosan/PAH compositions—100/0 (CTH-0), 80/10 (CTH-2), 60/40 (CTH-4), and 40/60 (CTH-5).

FTIR spectroscopy is a technique frequently employed to uncover intermolecular interactions, particularly hydrogen bonding between diverse types of functional groups in polymeric molecules. Intermolecular interactions either lead to a shift in frequency or band broadening of particular functional groups. Information related to the conformational changes occurring in compatible blends can be acquired by FTIR spectral analysis [65]. As we had prepared several solutions—CTH-0 (100/0), CTH-1 (90/10), CTH-2 (80/20), CTH-3 (70/30), CTH-4 (60/40), and CTH-5 (40/60)—of CS/PAH in different ratios, we investigated whether CS molecules really interacted with the polymeric backbone of PAH. To confirm possible interactions, the prepared biodegradable blend films—chitosan and PAH samples—were subjected to ATR-FTIR spectroscopy. The presence of each component was established by the appearance of absorption bands for different functional groups. The spectra of CS, PAH, and their blend films are shown in Figure 2. In the case of PAH spectra, the bands at 1610 cm^−1^ and 1381 cm^−1^ were detected corresponding to N–H in the plane bending motion and C–H bending, respectively [66]. The broadband varying from 3400 to 2800 cm^−1^ with two split peaks was observed—one peak at 2910 cm^−1^ and 2845 cm^−1^, attributed to C–H asymmetric and symmetric vibrations—while another sharp peak at 3391 cm^−1^ was caused by stretching vibration of the intermolecular as well as intramolecular N–H bond of the primary amine group. These peaks were observed in all blend films, but with less intensity [21].

In the spectra of chitosan, bands of amide-I (C=O) and amide-II (N–H) were detected at around 1640 cm^−1^ and 1538 cm^−1^, respectively. The cis-amide III band was located at 1248 cm^−1^ [62,67]. A peak at 1078 cm^−1^ confirmed C–O–C stretching vibrations [68]. The glycosidic linkages appeared at 1154 and 894 cm^−1^. Moreover, a band ranging from 3391 to 3386 cm^−1^ was assigned to the O–H stretching of chitosan [6,69]. We noticed that as the concentration of PAH in blend films increased, the intensity of the band also decreased. In the typical spectrum of a CS/PAH composite film, the amino peak of CS shifts from 1541 to 1510 cm^−1^ and the intensity of these bands decrease dramatically with the addition of PAH, indicating an intermolecular hydrogen bonding between CS and PAH (Figure 2) [16]. This manifests that CS has good compatibility with PAH.

### 3.3. Atomic Force Microscopy Study

**Figure 3:**
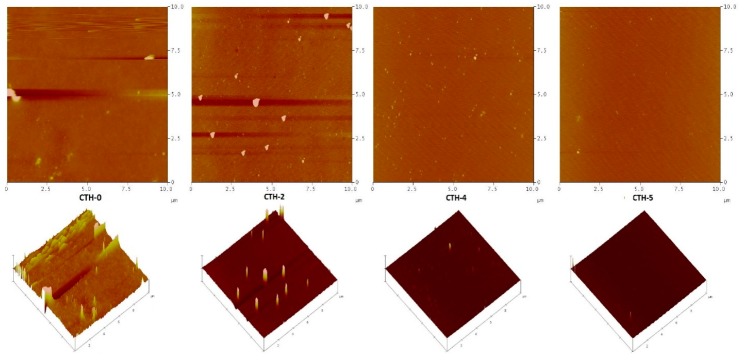
AFM images of films with different chitosan/PAH compositions—100/0 (CTH-0), 80/10 (CTH-2), 60/40 (CTH-4), and 40/60 (CTH-5)—and their corresponding 3D images in panels on mica using tapping mode. The scale size was 10 × 10 μm and the *z* scale was 20 nm and 30 nm, respectively.

AFM is an excellent tool that investigates the texture and morphology of diverse surfaces, including thin films [70,71,72]. The versatility of AFM allows acquiring of rigorous observations and assessment of the textural and morphological characteristics of films. In this study, the surface texture and roughness of CS and its blend films with PAH (CTH-0, CTH-1, CTH-2, CTH-3, CTH-4, CTH-5) were examined using AFM with a Nanoscope IIIa Multimode microscope (Veeco Instruments, Inc., Santa Barbara, CA, USA), equipped with a silicon RTESP7 cantilever (Veeco Nanoprobe, Camarillo, CA, USA) in tapping mode. For the silicon tip, nominal spring constant of 40 N/m was applied. AFM images were recorded as 10 μm × 10 μm using scan rate of 0.5 Hz under atmospheric conditions at ambient temperature. For AFM measurements, the blended solutions of polymers were spin-coated on a newly cleaved mica surface using a spin coater (Laurell Technologies Corporation, USA) and the spinning rate was maintained at 1500 RPM for 2 min. Experimental data were processed with the help of Nanoscope 5.30 software [13]. The micrographs infer that films coded with CTH-0 and CTH-2 were found to be rough. On the other hand, CTH-4 and CTH-5 films displayed a smooth surface morphology (Figure 3). This might be attributed to the rigid and crystalline nature of chitosan [73]. These properties of chitosan molecules were also affected by sample preparation. Like these films, CTH-0 and CTH-2 present spherical, branch crystals due to the higher percentage of chitosan 100% (*w*/*w*) and 80% (*w*/*w*), respectively [69]. AFM analysis showed that as the concentration of PAH increased up to 60% (*w/w*), the surface morphology improved, representing an increase in compatibility with polymer chains. The CTH-5 film was found to be more homogenous as compared to the other films.

### 3.4. WCA Measurements

**Figure 4:**
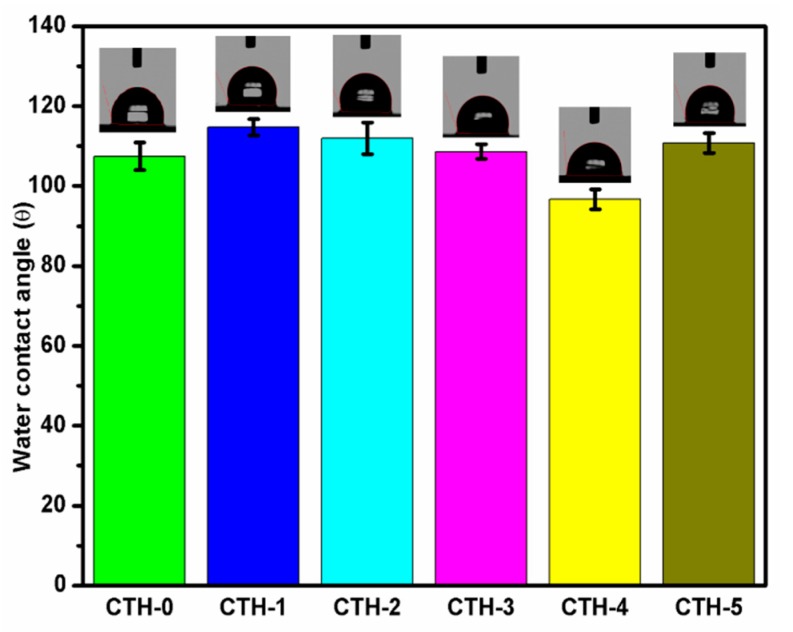
Water contact angles of films with chitosan/PAH ratios of 100/0 (CTH-0), 90/10 (CTH-1), 80/10 (CTH-2), 70/30 (CTH-3), 60/40 (CTH-4), and 40/60 (CTH-5).

Water contact angle measurement is one of the most common methodologies used to determine the wettability of a surface/material and their hydrophobic or hydrophilic nature [74]. The intrinsic values of WCA are dependent on surface roughness, porosity, and heterogeneity [75]. We recorded WCA of different images for each blend film using video contact angle instrument VCA optima (AST Products, Inc. Billerica, MA, USA) until WCA became constant. An automatic WCA measurement, from both the right and left-hand side of the drop, was done by VCA-optima software. On average, twenty measurements were performed per film, which was cut from four different sites. A report demonstrated that the water contact angle of blank chitosan was higher than 83° due to its hydrophilic nature and high surface tension [76]. In order to decrease the hydrophilicity of chitosan, it was blended with PAH to impart flexibility. The WCA of the prepared films fluctuated between 96.7° to 114.7° because of a CS-to-PAH ratio that varied as shown in Figure 4. As shown in Table 2, the WCA of CTH-0, CTH-1, CTH-2, CTH-3, CTH-4, and CTH-5 was 107 ± 3.5°, 114.7 ± 2.0°, 112 ± 3.9°, 108.6 ± 1.8°, 96.7 ± 2.5°, and 110.8 ± 2.5°, respectively. It was noted that as the concentration of PAH in blend films increased up to 60% (*w*/*w*), except 40% (*w*/*w*), the WCA increased. This increase might be due to a decrease in intensity of polar groups, resulting in more networking among polymer chains [9].

### 3.5. Swelling Behaviour in Different Media

#### 3.5.1. Swelling in Deionized Water

**Figure 5:**
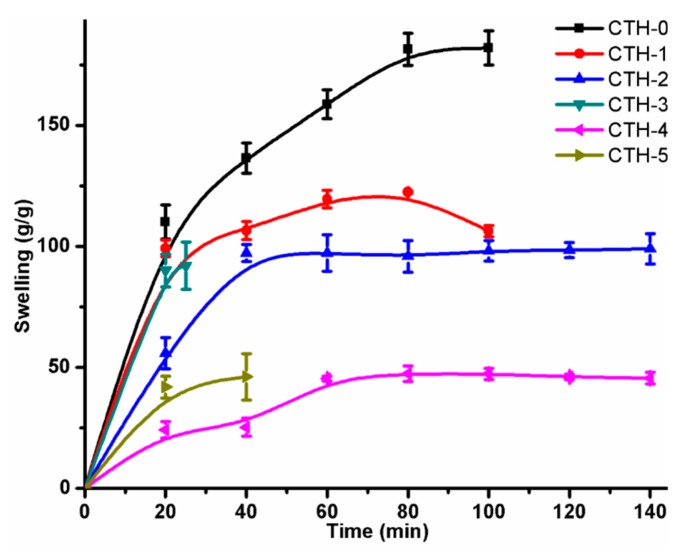
Swelling behaviour of CS/PAH films with different compositions in deionized water.

The swelling behaviour of pristine CS and CSPAH blend films was studied as a function of time (Table 3). It was observed that all films displayed a linear increase with time and equilibrium for the CTH-1, CTH-2, and CTH-4 films were attained at around 80 min, 60 min, and 80 min, respectively. The CTH-3 and CTH-5 films exhibited a distinctive behaviour as these films were converted into a gel-like material after 25 min and 40 min, respectively. The CTH-1 film had a higher degree of swelling response—around 122 g/g after 80 min—as compared to the other blend films, but less than blank chitosan film, which was around 182 g/g (Figure 5).

The swelling of blend films was due to diffusion of water from the extracellular matrix to the polymeric structures [77]. This mode of swelling was calculated by the following equation:

F = *kt^n^* (2)

where,

*k* = swelling rate constant describing the characteristics of polymer network and water 

*n* = swelling or diffusional exponent

*F* = fractional swelling at the time (*t*), calculated by the swelling ratio of *W*_t_ (swelling ratio at time *t*) and *W*_eq_ (swelling ratio at equilibrium time *t*).

**Figure 6:**
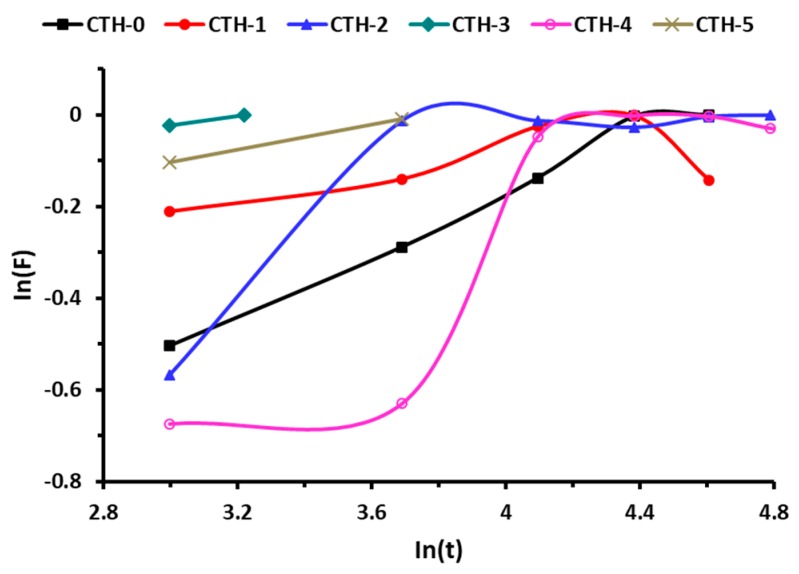
Swelling behavior of CS/PAH films with different compositions.

In order to obtain the values of *n* and *k*, the swelling data of pure CS and its blend films was used. Figure 6 showed a plot between ln(*F*) versus ln(*t*) and the values of diffusion parameters (Table 4). It can be inferred from Table 4 that *n* < 0.5 (corresponding to Fickian transport) for all films, which indicated that the rate of relaxation was higher than the rate of diffusion.

#### 3.5.2. Swelling Behaviour in Ionic Solutions

**Figure 7:**
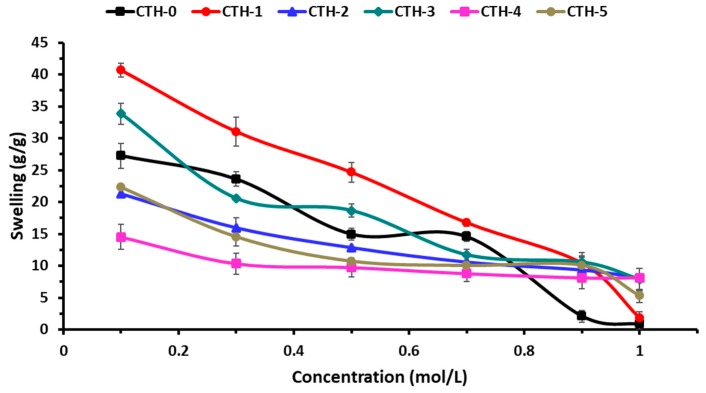
Swelling behavior of CS/PAH films in NaCl solutions of variable molar concentrations.

**Figure 8:**
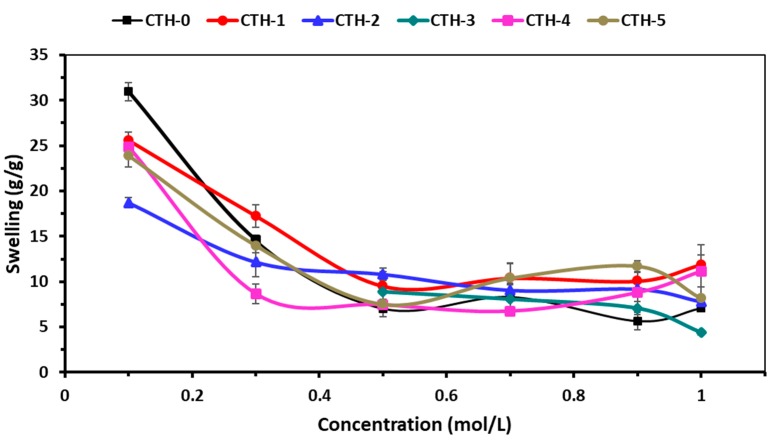
Swelling behaviour of CS/PAH films in different molar concentrations of CaCl_2_ solutions.

The swelling behaviour of pure CS and CS/PAH blend films as a function of time was evaluated in different ionic solutions, such as NaCl and CaCl_2_. The effect of the ionic concentration of electrolytes on the swelling behavior of blend films is shown in Figure 7 and Figure 8. These Figures demonstrate that with the increase of concentration of electrolyte in swelling medium, the swelling capacity of blend films significantly decreased. It was found that the nature of salt and the concentration of ions had a noticeable influence on the swelling response. As the concentration of both electrolytes (NaCl and CaCl_2_) increased, the rate of swelling decreased (Table 5 and Table 6). This might be due to the generation of high-charge screening effects, which ultimately reduced the rate of diffusion and osmotic pressure developed between the films and the external solvent. Consequently, the diffusion of solvent into blend film decreased, resulting in lesser swelling. From Figure 7 and Figure 8, it can be inferred that maximum swelling was shown by CTH-1 in NaCl (0.1 M) compared to CaCl_2_ (0.1 M). The swelling value for CTH-1 was 41 g/g and 26 g/g in NaCl (0.1 M) and CaCl_2_ (0.1 M), respectively [77]. Furthermore, overall higher swelling response was noticed in NaCl solution, as compared to other CaCl_2_ solutions. It was observed that the CTH-3 film was converted into gel-like material in 0.1 M and 0.3 M CaCl_2_ solutions.

### 3.6. Cumulative Drug Release (CDR) Study

**Figure 9:**
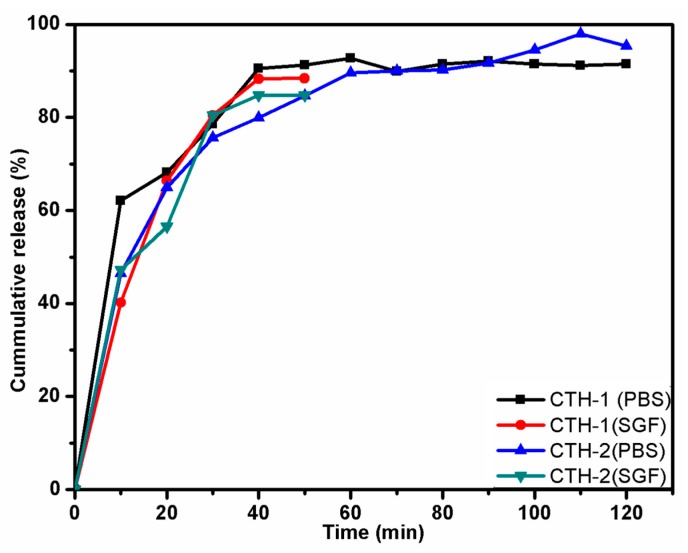
Data on cumulative drug release (%) in SGF and PBS solutions.

Ciprofloxacin hydrochloride monohydrate, a model drug, was loaded in two films coded as CTH-1 and CTH-2 (Table 7). Based on encouraging results as described in Section 3.1, Section 3.2, Section 3.3, Section 3.4 and Section 3.5, the CTH-1 and CTH-2 blend films were selected. These two blend films had a greater concentration of natural polymer used in the present study (chitosan). However, CTH-3 and CTH-5 showed more solubility in aqueous media and CTH-4 film had a greater concentration of PAH. The controlled drug release from both films (CTH-1 and CTH-2) was studied in SGF (pH 1.2) as well as in PBS (pH 7.4) solution as a function of time at 37 °C (Figure 9). In the case of SGF, it was observed that most of the drug, around 88% from CTH-1 and 85% from CTH-2, was released in 50 min. On the contrary, in the PBS solution, the CDR for CTH-1 and CTH-2 was around 93% in 90 min and 98% in 120 min, respectively. The balance amount of drug could not be determined because both films converted into gel-like material due to acidic dissolution in SGF and fragmented in PBS after 30 min (CTH-1) and 120 min (CTH-1). The drug release in PBS solution at 120 min for CTH-1 sample was significantly higher in the CTH-2 sample (mean = 95.30%, SD = 1.40) than the CTH-1 sample (mean = 91.44%, SD = 1.87), *t* (4) = −2.86, *p*-value < 0.05. A similar pattern was observed for the SGF solution; mean for the CTH-1 sample was 88.39% (SD = 0.51), while the mean was 84.78% (SD = 0.48) for the CTH-2 sample; *t* (4) = 8.87, *p*-value < 0.005. From these results, it could be deduced that the drug was released in a controlled manner in the PBS solution, when compared to the SGF.

## 4. Conclusions

In this study, a drug delivery matrix based on chitosan/PAH blend films with different compositions was successfully prepared by the solution casting method and their physicochemical characteristics were evaluated. These films were found to have a homogenous architecture and excellent surface morphology. AFM exhibited improvements in surface morphology when the PAH concentration was increased up to 60% (*w/w*). CTH-1 (90% chitosan and 10% PAH) showed maximum WCA, which was 115°. Moreover, the CS/PAH blend films exhibited varied swelling responses in different media and sustained drug release behaviour in PBS, as compared to SGF. The swelling response of blend films in deionized water showed a linear increase as a function of time, and CTH-1 (90% CS and 10% PAH) had a higher degree of swelling, which was around 122 g/g after 80 min, as compared to the other blend films. The maximum observed swelling value in NaCl (0.1 M) for CTH-1 was 41 g/g and in CaCl_2_ (0.1 M) 31 g/g for CTH-0 (100% CS). Two films were selected, CTH-1 (90% CS and 10% PAH) and CTH-2 (80% CS and 20% PAH), for in vitro CDR at 37 °C. In SGF (pH 1.2), the CDR for CTH-1 and CTH-2 was about 88% and 85% in 50 min, respectively. In SGF, both films were converted into gel-like material after 30 min due to lesser stability of films at lower pH. In the PBS solution (pH 7.4), meanwhile, the CDR for CTH-1 and CTH-2 was around 93% in 90 min and 98% in 120 min, respectively. It can be concluded that the drug was released in a controlled manner in the PBS solution, as compared to the SGF. The selected blend films showed a relatively reduced release (CHT1 = 88% in 50 min, CTH2 = 85% 50 min) in simulated gastric fluid (pH = 1.2) and maximum drug release (CHT1 = 93% in 90 min, CTH2 = 98% 120 min) in phosphate buffer saline (pH = 7.4), thus indicating a pH-responsive (smart) nature. Although the CTH-1 and CTH-2 films showed remarkable drug release, they converted to a gel-like material over a period of time (30 min) in acidic pH (pH = 1.2). This showed the pH-responsive nature of CTH-1 and CTH2. In summary, the results show that chitosan CS/PAH are excellent drug delivery matrices for drug release at pH 7.4. These blend films can be employed for injectable drug delivery systems, tissue regeneration, and associated biomedical applications such as wound dressing due to good compatibility between the drug and the film matrix; they may, however, may not be suitable candidates for oral drug administration.

## Figures and Tables

**Table 1:** Films thickness data of each film from different regions.

Films	Films Thickness (1st region) (µm)	Films Thickness (2nd region) (µm)	Films Thickness (3rd region) (µm)	Films Thickness (4th region) (µm)	Average Films Thickness (µm)	* SD (±)
CTH-0	40	50	70	50	52.5	10.9
CTH-1	70	80	90	40	70	18.7
CTH-2	40	40	50	60	47.5	8.3
CTH-3	50	70	40	50	52.5	10.9
CTH-4	70	70	40	70	62.5	13.0
CTH-5	50	40	30	40	40	7.1

* SD means standard deviation, CTH stands for chitosan/poly(allylamine hydrochloride) blend.

**Table 2:** Water contact angles data of films having CS/PAH ratios of 100/0 (CTH-0), 90/10 (CTH-1), 80/10 (CTH-2), 70/30 (CTH-3), 60/40 (CTH-4), and 40/60 (CTH-5).

Films	Water Contact Angle (degree)	* SD (±)
CTH-0	107.4	3.5
CTH-1	114.7	2.0
CTH-2	112.0	3.9
CTH-3	108.6	1.8
CTH-4	96.7	2.5
CTH-5	110.8	2.5

* SD means standard deviation; all experiments were performed in triplicate.

**Table 3:** Swelling data of CS/PAH films in deionized water.

Time (min)	CTH-0		CTH-1		CTH-2		CTH-3		CTH-4		CTH-5	
	Swelling (g/g)	SD (±)	Swelling (g/g)	SD (±)	Swelling (g/g)	SD (±)	Swelling (g/g)	SD (±)	Swelling (g/g)	SD (±)	Swelling (g/g)	SD (±)
20	109.96	7.06	99.15	3.49	55.90	6.42	90.00	6.70	24.20	3.43	41.90	4.56
40	136.41	6.25	106.49	3.71	97.26	3.59	92.00	9.70	25.30	3.77	46.12	9.59
60	158.62	5.95	119.52	3.67	97.27	7.51	--	--	45.32	1.04	--	--
80	181.39	6.70	122.40	0.57	95.86	6.52	--	--	47.42	3.21	--	--
100	181.95	7.08	106.29	2.27	98.14	4.18	--	--	47.35	2.33	--	--
120	--	--	--	--	98.44	3.19	--	--	46.14	1.09	--	--
140	--	--	--	--	98.99	6.22	--	--	45.59	2.46	--	--

SD means standard deviation; in order to obtain standard deviation, the experiment was repeated three times for each film.

**Table 4:** Diffusion parameters of different CS/PAH films.

Parameters	CTH-0	CTH-1	CTH-2	CTH-3	CTH-4	CTH-5
*n*	0.355	0.160	0.280	0.098	0.442	0.138
Intercept	−1.58	−0.70	−1.25	−0.31	−2.040	−0.51
*k*	0.457	−0.357	0.223	−1.204	0.713	−0.673

The experiment was repeated three times for each film.

**Table 5:** Data showing swelling behaviour of CS/PAH films in different molar concentrations of NaCl.

[NaCl]	CTH-0		CTH-1		CTH-2		CTH-3		CTH-4		CTH-5	
	Swelling (g/g)	SD (±)	Swelling (g/g)	SD (±)	Swelling (g/g)	SD (±)	Swelling (g/g)	SD (±)	Swelling (g/g)	SD (±)	Swelling (g/g)	SD (±)
0.1 M	27.25	1.95	40.69	1.09	21.32	0.00	33.84	1.62	14.53	1.96	22.33	0.23
0.3 M	23.58	1.14	31.04	2.28	15.98	1.53	20.56	0.07	10.33	1.69	14.54	1.48
0.5 M	14.95	0.95	24.66	1.51	12.86	0.07	18.65	1.04	9.74	1.52	10.70	0.38
0.7 M	14.56	0.76	16.75	0.52	10.59	1.95	11.70	0.93	8.78	1.24	10.03	0.50
0.9 M	2.11	0.92	10.38	1.22	9.34	1.40	10.56	1.49	8.12	1.73	10.07	1.24
1.0 M	0.84	0.83	1.79	1.02	8.10	0.59	7.85	1.74	8.14	0.17	5.27	1.07

SD means standard deviation; in order to obtain standard deviation, the experiment was performed in triplicate for each film.

**Table 6:** Data showing swelling behaviour of CS/PAH films in CaCl_2_ solutions of variable molar concentrations.

[CaCl_2_]	CTH-0		CTH-1		CTH-2		CTH-3		CTH-4		CTH-5	
	Swelling (g/g)	SD (±)	Swelling (g/g)	SD (±)	Swelling (g/g)	SD (±)	Swelling (g/g)	SD (±)	Swelling (g/g)	SD (±)	Swelling (g/g)	SD (±)
0.1 M	30.95	0.99	25.54	0.99	18.69	0.56	-	-	24.83	0.84	23.87	1.21
0.3 M	14.55	0.49	17.22	1.26	12.16	1.63	-	-	8.66	1.06	13.96	0.74
0.5 M	7.04	0.90	9.50	1.59	10.82	0.69	8.91	1.69	7.48	0.15	7.51	0.56
0.7 M	8.33	1.50	10.38	1.59	9.06	0.57	8.10	0.01	6.76	0.37	10.40	1.63
0.9 M	5.67	0.98	10.06	0.97	9.18	1.89	7.09	0.75	8.82	0.18	11.70	0.62
1.0 M	7.11	0.25	11.88	1.07	7.80	0.68	4.42	0.26	11.17	2.86	8.15	1.23

SD means standard deviation; in order to obtain standard deviation, the experiment was repeated three times for each film.

**Table 7:** Data on cumulative drug release (%) in SGF and PBS solutions.

Time (min)	CTH-1 (PBS)%	* SD (±)	CTH-1 (SGF)%	* SD (±)	CTH-2 (PBS)%	SD (±)	CTH-2 (SGF)%	SD (±)
0	0.00	0.00	0.00	0.00	0.00	0.00	0.00	0.00
10	62.11	0.90	40.21	0.30	46.47	0.50	47.21	0.30
20	68.19	1.00	66.42	0.50	64.95	0.60	56.56	0.50
30	78.55	1.20	80.45	0.30	75.60	0.40	80.45	0.30
40	90.50	1.50	88.26	0.30	79.88	0.70	84.74	0.30
50	91.30	0.90	88.39	0.60	84.63	0.10	84.80	0.60
60	92.73	2.00	--	--	89.59	0.30	--	--
70	89.90	2.00	--	--	89.99	0.50	--	--
80	91.46	1.80	--	--	90.19	0.30	--	--
90	92.09	1.80	--	--	91.64	0.30	--	--
100	91.49	1.60	--	--	94.49	0.60	--	--
110	91.13	1.20	--	--	97.91	0.20	--	--
120	91.44	0.60	--	--	95.31	0.60	--	--

* SD means standard deviation; in order to obtain standard deviation, the experiment was repeated three times for each film.

## References

[1] Gazori T., Khoshayand M.R., Azizi E., Yazdizade P., Nomani A., Haririan I. (2009). Evaluation of Alginate/Chitosan nanoparticles as antisense delivery vector: Formulation, optimization and in vitro characterization. Carbohydr. Polym..

[2] Gemma V., Judit T.-P., Fernando A. (2012). Polymers and Drug Delivery Systems. Curr. Drug Deliv..

[3] Dan Mogoşanu G., Mihai Grumezescu A., Everard Bejenaru L., Bejenaru C., Grumezescu A.M. (2016). Chapter 8—Natural and synthetic polymers for drug delivery and targeting. Nanobiomaterials in Drug Delivery.

[4] Niaz T., Nasir H., Shabbir S., Rehman A., Imran M. (2016). Polyionic hybrid nano-engineered systems comprising alginate and chitosan for antihypertensive therapeutics. Int. J. Biol. Macromol..

[5] Cong Z., Shi Y., Wang Y., Wang Y., Chen N., Xue H. (2018). A novel controlled drug delivery system based on alginate hydrogel/chitosan micelle composites. Int. J. Biol. Macromol..

[6] Costa-Júnior E.S., Barbosa-Stancioli E.F., Mansur A.A.P., Vasconcelos W.L., Mansur H.S. (2009). Preparation and characterization of chitosan/poly(vinyl alcohol) chemically crosslinked blends for biomedical applications. Carbohydr. Polym..

[7] Husain S., Al-Samadani K.H., Najeeb S., Zafar M.S., Khurshid Z., Zohaib S., Qasim S.B. (2017). Chitosan Biomaterials for Current and Potential Dental Applications. Materials.

[8] Qasim S.B., Zafar M.S., Najeeb S., Khurshid Z., Shah A.H., Husain S., Rehman I.U. (2018). Electrospinning of Chitosan-Based Solutions for Tissue Engineering and Regenerative Medicine. Int. J. Mol. Sci..

[9] Nady N., Kandil S.H. (2018). Novel Blend for Producing Porous Chitosan-Based Films Suitable for Biomedical Applications. Membranes.

[10] Seo D.-H., Jeong Y.-I., Kim D.-G., Jang M.-J., Jang M.-K., Nah J.-W. (2009). Methotrexate-incorporated polymeric nanoparticles of methoxy poly(ethylene glycol)-grafted chitosan. Colloids Surf. B Biointerfaces.

[11] Szymanska E., Winnicka K. (2015). Stability of chitosan-a challenge for pharmaceutical and biomedical applications. Mar. Drugs.

[12] Sarasam A.R., Krishnaswamy R.K., Madihally S.V. (2006). Blending Chitosan with Polycaprolactone:  Effects on Physicochemical and Antibacterial Properties. Biomacromolecules.

[13] Yang X., Tu Y., Li L., Shang S., Tao X.-m. (2010). Well-dispersed chitosan/graphene oxide nanocomposites. ACS Appl. Mater. Interfaces.

[14] Doulabi A.H., Mirzadeh H., Imani M., Samadi N. (2013). Chitosan/polyethylene glycol fumarate blend film: Physical and antibacterial properties. Carbohydr. Polym..

[15] Lanjhiyana S.K., Bajpayee P., Kesavan K., Lanjhiyana S., Muthu M.S. (2013). Chitosan–sodium alginate blended polyelectrolyte complexes as potential multiparticulate carrier system: Colon-targeted delivery and gamma scintigraphic imaging. Expert Opin. Drug Deliv..

[16] Pulieri E., Chiono V., Ciardelli G., Vozzi G., Ahluwalia A., Domenici C., Vozzi F., Giusti P. (2008). Chitosan/gelatin blends for biomedical applications. J. Biomed. Mater. Res. Part A.

[17] Damodharan N., Krishnamoorthy B., Kumar B.S., Soundrapandian C. (2008). Chitosan-polyethylene glycol blend films as local ophthalmic drug delivery units: In vitro studies and factors influencing drug release. Asian J. Chem..

[18] Kamoun E.A. (2016). N-succinyl chitosan–dialdehyde starch hybrid hydrogels for biomedical applications. J. Adv. Res..

[19] Khan M.I.H., An X., Dai L., Li H., Khan A., Ni Y. (2019). Chitosan-based Polymer Matrix for Pharmaceutical Excipients and Drug Delivery. Curr. Med. Chem..

[20] Sarwar M.S., Ghaffar A., Islam A., Yasmin F., Oluz Z., Tuncel E., Duran H., Qaiser A.A. (2020). Controlled drug release behavior of metformin hydrogen chloride from biodegradable films based on chitosan/poly(ethylene glycol) methyl ether blend. Arab. J. Chem..

[21] Deshmukh K., Ahmad J., Hägg M.B. (2014). Fabrication and characterization of polymer blends consisting of cationic polyallylamine and anionic polyvinyl alcohol. Ionics.

[22] Jian W., Xu S., Wang J., Feng S. (2013). Layer-by-layer assembly of poly(allylamine hydrochloride)/polyurethane and its loading and release behavior for methylene orange. J. Appl. Polym. Sci..

[23] Kreke M.R., Badami A.S., Brady J.B., Akers R.M., Goldstein A.S. (2005). Modulation of protein adsorption and cell adhesion by poly(allylamine hydrochloride) heparin films. Biomaterials.

[24] Bhat S., Tripathi A., Kumar A. (2011). Supermacroprous chitosan-agarose-gelatin cryogels: In vitro characterization and in vivo assessment for cartilage tissue engineering. J. R. Soc. Interface.

[25] Miguel S.P., Ribeiro M.P., Brancal H., Coutinho P., Correia I.J. (2014). Thermoresponsive chitosan-agarose hydrogel for skin regeneration. Carbohydr. Polym..

[26] Quiroz-Castillo J.M., Rodriguez-Felix D.E., Grijalva-Monteverde H., Del Castillo-Castro T., Plascencia-Jatomea M., Rodriguez-Felix F., Herrera-Franco P.J. (2014). Preparation of extruded polyethylene/chitosan blends compatibilized with polyethylene-graft-maleic anhydride. Carbohydr. Polym..

[27] Belluzo M.S., Medina L.F., Cortizo A.M., Cortizo M.S. (2016). Ultrasonic compatibilization of polyelectrolyte complex based on polysaccharides for biomedical applications. Ultrason. Sonochemistry.

[28] Zarrintaj P., Saeb M.R., Jafari S.H., Mozafari M., Ajitha A.R., Thomas S. (2020). Chapter 18—Application of compatibilized polymer blends in biomedical fields. Compatibilization of Polymer Blends.

[29] Shanavas A., Bahadur D., Srivastava R. Core/surface modified nanomedicines for controlled release of drug. Proceedings of the 12th IEEE Conference on Nanotechnology (IEEE-NANO).

[30] Luo R., Neu B., Venkatraman S.S. (2012). Surface functionalization of nanoparticles to control cell interactions and drug release. Small.

[31] Ueno H., Mori T., Fujinaga T. (2001). Topical formulations and wound healing applications of chitosan. Adv. Drug Deliv. Rev..

[32] Dash M., Chiellini F., Ottenbrite R.M., Chiellini E. (2011). Chitosan—A versatile semi-synthetic polymer in biomedical applications. Prog. Polym. Sci..

[33] Uhrich K.E., Cannizzaro S.M., Langer R.S., Shakesheff K.M. (1999). Polymeric Systems for Controlled Drug Release. Chem. Rev..

[34] Gupta K.C., Ravi Kumar M.N.V. (2000). Drug release behavior of beads and microgranules of chitosan. Biomaterials.

[35] Jin J., Song M. (2006). Chitosan and chitosan–PEO blend membranes crosslinked by genipin for drug release. J. Appl. Polym. Sci..

[36] Chitosan-Based Drug Delivery Systems. Chitin and Chitosan.

[37] Wang L.-C., Chen X.-G., Zhong D.-Y., Xu Q.-C. (2007). Study on poly(vinyl alcohol)/carboxymethyl-chitosan blend film as local drug delivery system. J. Mater. Sci.: Mater. Med..

[38] Mathew S., Brahmakumar M., Abraham T.E. (2006). Microstructural imaging and characterization of the mechanical, chemical, thermal, and swelling properties of starch-chitosan blend films. Biopolymers.

[39] Liu C., Xiao C. (2004). Characterization of films from chitosan and quaternized poly(4-vinyl-*N*-butyl) pyridine solutions. J. Appl. Polym. Sci..

[40] Teodorescu M., Bercea M., Morariu S. (2018). Biomaterials of Poly(vinyl alcohol) and Natural Polymers. Polym. Rev..

[41] Shieh J.-J., Huang R.Y.M. (1998). Chitosan/N-methylol nylon 6 blend membranes for the pervaporation separation of ethanol–water mixtures. J. Membr. Sci..

[42] Tanabe T., Okitsu N., Tachibana A., Yamauchi K. (2002). Preparation and characterization of keratin–chitosan composite film. Biomaterials.

[43] Xu Y.X., Kim K.M., Hanna M.A., Nag D. (2005). Chitosan–starch composite film: Preparation and characterization. Ind. Crop. Prod..

[44] Yin J., Luo K., Chen X., Khutoryanskiy V.V. (2006). Miscibility studies of the blends of chitosan with some cellulose ethers. Carbohydr. Polym..

[45] Alexeev V.L., Kelberg E.A., Evmenenko G.A., Bronnikov S.V. (2000). Improvement of the mechanical properties of chitosan films by the addition of poly(ethylene oxide). Polym. Eng. Sci..

[46] Tsai G.-J., Zhang S.-L., Shieh P.-L. (2004). Antimicrobial Activity of a Low-Molecular-Weight Chitosan Obtained from Cellulase Digestion of Chitosan. J. Food Prot..

[47] Sébastien F., Stéphane G., Copinet A., Coma V. (2006). Novel biodegradable films made from chitosan and poly(lactic acid) with antifungal properties against mycotoxinogen strains. Carbohydr. Polym..

[48] Ghaffar A., Schoenmakers P.J., van der Wal S. (2014). Methods for the chemical analysis of degradable synthetic polymeric biomaterials. Crit. Rev. Anal. Chem..

[49] Atta S., Khaliq S., Islam A., Javeria I., Jamil T., Athar M.M., Shafiq M.I., Ghaffar A. (2015). Injectable biopolymer based hydrogels for drug delivery applications. Int. J. Biol. Macromol..

[50] Blondeau J.M. (2004). Fluoroquinolones: Mechanism of action, classification, and development of resistance. Surv. Ophthalmol..

[51] Wilhelmus K.R., Hyndiuk R.A., Caldwell D.R., Abshire R.L., Folkens A.T., Godio L.B. (1993). 0.3% ciprofloxacin ophthalmic ointment in the treatment of bacterial keratitis. Arch. Ophthalmol..

[52] Acar J., Goldstein F. (1997). Trends in bacterial resistance to fluoroquinolones. Clin. Infect. Dis..

[53] Huneault L.M., Lussier B., Dubreuil P., Chouinard L., Désévaux C. (2004). Prevention and treatment of experimental osteomyelitis in dogs with ciprofloxacin-loaded crosslinked high amylose starch implants. J. Orthop. Res..

[54] Mehnert W., Mäder K. (2001). Solid lipid nanoparticles: Production, characterization and applications. Adv. Drug Deliv. Rev..

[55] Segev S., Yaniv I., Haverstock D., Reinhart H. (1999). Safety of long-term therapy with ciprofloxacin: Data analysis of controlled clinical trials and review. Clin. Infect. Dis. Off. Publ. Infect. Dis. Soc. Am..

[56] Bader M.S., Hawboldt J., Brooks A. (2010). Management of complicated urinary tract infections in the era of antimicrobial resistance. Postgrad. Med..

[57] Tsou T.L., Tang S.T., Huang Y.C., Wu J.R., Young J.J., Wang H.J. (2005). Poly(2-hydroxyethyl methacrylate) wound dressing containing ciprofloxacin and its drug release studies. J. Mater. Sci. Mater. Med..

[58] Unnithan A.R., Barakat N.A., Pichiah P.B., Gnanasekaran G., Nirmala R., Cha Y.S., Jung C.H., El-Newehy M., Kim H.Y. (2012). Wound-dressing materials with antibacterial activity from electrospun polyurethane-dextran nanofiber mats containing ciprofloxacin HCl. Carbohydr. Polym..

[59] Ajmal G., Bonde G.V., Thokala S., Mittal P., Khan G., Singh J., Pandey V.K., Mishra B. (2019). Ciprofloxacin HCl and quercetin functionalized electrospun nanofiber membrane: Fabrication and its evaluation in full thickness wound healing. Artif. Cells Nanomed. Biotechnol..

[60] Kioomars S., Heidari S., Malaekeh-Nikouei B., Shayani Rad M., Khameneh B., Mohajeri S.A. (2017). Ciprofloxacin-imprinted hydrogels for drug sustained release in aqueous media. Pharm. Dev. Technol..

[61] Zhang M., Li X., Gong Y., Zhao N., Zhang X. (2002). Properties and biocompatibility of chitosan films modified by blending with PEG. Biomaterials.

[62] Jabeen S., Islam A., Ghaffar A., Gull N., Hameed A., Bashir A., Jamil T., Hussain T. (2017). Development of a novel pH sensitive silane crosslinked injectable hydrogel for controlled release of neomycin sulfate. Int. J. Biol. Macromol..

[63] Sambrook J., Fritsch E., Maniatis T. (1989). Molecular Cloning: A Laboratory Manual 1989 2nd edn Cold Spring Harbor. NY Cold Spring Harb. Lab..

[64] Wang Q., Zhang J., Wang A. (2009). Preparation and characterization of a novel pH-sensitive chitosan-*g*-poly(acrylic acid)/attapulgite/sodium alginate composite hydrogel bead for controlled release of diclofenac sodium. Carbohydr. Polym..

[65] Lu L., Peng F., Jiang Z., Wang J. (2006). Poly(vinyl alcohol)/chitosan blend membranes for pervaporation of benzene/cyclohexane mixtures. J. Appl. Polym. Sci..

[66] Mathapa B.G., Paunov V.N. (2013). Fabrication of novel cyclodextrin-polyallylamine hydrochloride co-polymeric microcapsules by templating oil-in-water emulsions. Soft Matter.

[67] Wang Q., Dong Z., Du Y., Kennedy J.F. (2007). Controlled release of ciprofloxacin hydrochloride from chitosan/polyethylene glycol blend films. Carbohydr. Polym..

[68] Gregorio-Jauregui K.M., Pineda M.G., Rivera-Salinas J.E., Hurtado G., Saade H., Martinez J.L., Ilyina A., López R.G. (2012). One-step method for preparation of magnetic nanoparticles coated with chitosan. J. Nanomater..

[69] He L.-H., Xue R., Yang D.-B., Liu Y., Song R. (2009). Effects of blending chitosan with peg on surface morphology, crystallization and thermal properties. Chin. J. Polym. Sci..

[70] Kumar B.R., Rao T.S. (2012). AFM Studies on surface morphology, topography and texture of nanostructured zinc aluminum oxide thin films. Dig. J. Nanomater. Biostructures.

[71] Nagabhushana K.R., Lakshminarasappa B.N., Narasimha Rao K., Singh F., Sulania I. (2008). AFM and photoluminescence studies of swift heavy ion induced nanostructured aluminum oxide thin films. Nucl. Instrum. Methods Phys. Res. Sect. B Beam Interact. Mater. At..

[72] Kwoka M., Ottaviano L., Szuber J. (2007). AFM study of the surface morphology of L-CVD SnO_2_ thin films. Thin Solid Film.

[73] Croisier F., Jérôme C. (2013). Chitosan-based biomaterials for tissue engineering. Eur. Polym. J..

[74] Dwivedi C., Pandey I., Pandey H., Ramteke P.W., Pandey A.C., Mishra S.B., Patil S., Grumezescu A.M. (2017). Chapter 9—Electrospun Nanofibrous Scaffold as a Potential Carrier of Antimicrobial Therapeutics for Diabetic Wound Healing and Tissue Regeneration. Nano- and Microscale Drug Delivery Systems.

[75] Drioli E., Criscuoli A., Curcio E. (2006). Chapter 2—Membrane materials. Membrane Science and Technology.

[76] Ma G., Yang D., Su D., Mu X., Kennedy J.F., Nie J. (2010). Preparation and properties of water-soluble chitosan and polyvinyl alcohol blend films as potential bone tissue engineering matrix. Polym. Adv. Technol..

[77] Islam A., Yasin T. (2012). Controlled delivery of drug from pH sensitive chitosan/poly(vinyl alcohol) blend. Carbohydr. Polym..

